# Evaluation of changes in drug susceptibility and population genetic structure in *Haemonchus contortus* following worm replacement as a means to reverse the impact of multiple-anthelmintic resistance on a sheep farm

**DOI:** 10.1016/j.ijpddr.2021.02.004

**Published:** 2021-02-18

**Authors:** Melissa M. George, Adriano F. Vatta, Sue B. Howell, Bob E. Storey, Ciaran J. McCoy, Adrian J. Wolstenholme, Elizabeth M. Redman, John S. Gilleard, Ray M. Kaplan

**Affiliations:** a Department of Infectious Diseases, College of Veterinary Medicine, University of Georgia, 501 D.W. Brooks Drive, Athens, GA, 30602, USA; b Department of Comparative Biology and Experimental Medicine, Faculty of Veterinary Medicine, University of Calgary, 3330, Hospital Drive, Calgary, Alberta, T2N 4N1, Canada

**Keywords:** *Haemonchus*, Resistance, Replacement, Sheep, Genetic

## Abstract

A population of *Haemonchus contortus* that was highly resistant to benzimidazoles and avermectin/milbemycins with a subpopulation that was resistant to levamisole, was replaced with a susceptible laboratory isolate of *H. contortus* in a flock of sheep*.* The anthelmintic susceptibility and population genetics of the newly established population were evaluated for 3.5 years using *in vivo*, *in vitro*, and molecular methods. Successful replacement of the resistant population with a susceptible population was confirmed using phenotypic and genotypic measurements; larval development assay indicated full anthelmintic susceptibility; albendazole treatment yielded 98.7% fecal egg count reduction; pyrosequence genotyping of single nucleotide polymorphisms in positions 167 and 200 of the isotype-1 beta tubulin gene were present at 0.0 and 1.7%, respectively; microsatellite genotyping indicated the background haplotype was similar to the susceptible isolate; and haplotypes of the isotype-1 beta tubulin gene were similar to the susceptible isolate. To sustain the susceptibility of the new population, targeted selective treatment was implemented using albendazole. Surprisingly, within 1.5 years post-replacement, the population reverted to a resistant phenotype. Resistance to albendazole, ivermectin, and moxidectin was confirmed via fecal egg count reduction test, larval development assay, and pyrosequencing-based genotyping. Targeted selective treatment was then carried out using levamisole. However, within one year, resistance was detected to levamisole. Population genetics demonstrated a gradual change in the genetic structure of the population until the final population was similar to the initial resistant population. Genetic analyses showed a lack of diversity in the susceptible isolate, suggesting the susceptible isolate had reduced environmental fitness compared to the resistant population, providing a possible explanation for the rapid reversion to resistance. This work demonstrates the power of combining molecular, *in vitro*, and *in vivo* assays to study phenotypic and genotypic changes in a field population of nematodes, enabling improved insights into the epidemiology of anthelmintic resistance.

## Graphical abstract

**Figure:**
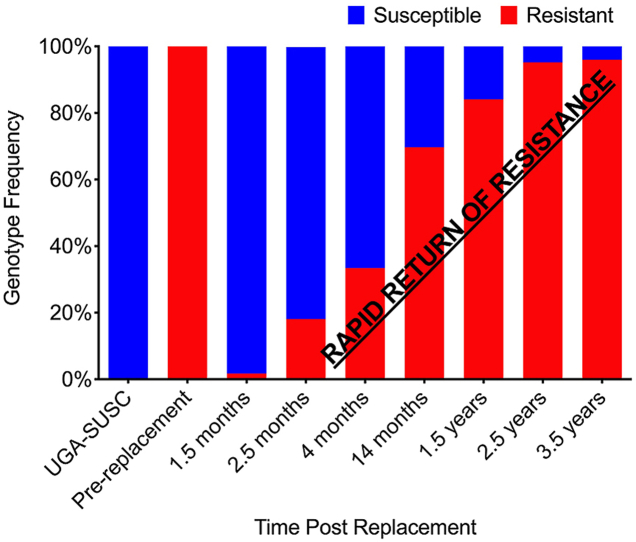

## 1 Introduction

The highly pathogenic parasitic nematode *Haemonchus contortus* has a remarkably high propensity to develop drug resistance (Gilleard and Devaney, 2013). Anthelmintic resistance to multiple classes of drugs is a serious problem on small ruminant farms across the globe (Howell et al., 2008; Torres-Acosta et al., 2012; Playford et al., 2014), providing a significant challenge to effective parasite control (Kaplan and Vidyashankar, 2012). To slow the development of anthelmintic resistance, Leathwick and Besier (2014) suggest producers implement refugia-based control strategies, use highly-effective combinations of anthelmintics, and prevent the introduction of resistant nematodes. Though implementation of these strategies will slow the development of resistance, the prevalence of farms with multiple-anthelmintic resistance and total anthelmintic failure is already high and is increasing (Martínez-Valladares et al., 2013; Herrera-Manzanilla et al., 2017; Howell et al., 2017). Thus, the aforementioned preventative strategies to slow the development of resistance are no longer useful or practical on many of these properties (Leathwick et al., 2012), leaving few options for chemical-based control and necessitating the development of novel approaches. Hence, replacement of a resistant population with a susceptible population (Van Wyk and Van Schalkwyk, 1990) may be an attractive strategy for properties where the parasite population is already resistant to multiple classes of anthelmintics (Leathwick et al., 2015). Replacement with a susceptible population of gastrointestinal nematodes would allow producers to reclaim the use of classes of anthelmintics that were previously ineffective. If then used in conjunction with sustainable integrated parasite management (sIPM) strategies (van Wyk et al., 2006; Leathwick and Besier, 2014), this approach could serve as a long-term solution for these farms by maintaining the effectiveness of the anthelmintics into the future.

Replacement of a resistant population of gastrointestinal nematodes with a susceptible population is a strategy designed to augment the population's genetic structure and replace resistant alleles with susceptible alleles (Van Wyk and Van Schalkwyk, 1990). Replacement was previously tested in gastrointestinal nematodes of sheep with various methodologies for introduction of susceptible parasites (Van Wyk and Van Schalkwyk, 1990; Bird et al., 2001; Aumont, 2002; Sissay et al., 2006; Moussavou-Boussougou et al., 2007; Muchiut et al., 2019). Level of drug efficacy following replacement varied widely among these studies depending on the strategy used to reduce the resistant population, the methodology to introduce the susceptible population, the season the study was conducted, and the period of time post-replacement that fecal egg count reduction (FECR) was evaluated (Muchiut et al., 2018). Previously published evaluations of replacement measured changes in susceptibility at points in time that were less than 18 months from replacement (Van Wyk and Van Schalkwyk, 1990; Bird et al., 2001; Aumont, 2002; Sissay et al., 2006; Moussavou-Boussougou et al., 2007; Muchiut et al., 2019). Eighteen months likely is not a sufficient period in most cases to determine if the observed short-term changes in susceptibility are sustainable long-term.

In this study we used multiple phenotypic and genotypic approaches to examine changes in the drug susceptibility and population genetics of the replaced *H. contortus* population. For measuring the resistance phenotype, we used both *in vivo* (fecal egg count reduction test, FECRT) and *in vitro* (Larval Development Assay) measures, and for the resistance genotype for benzimidazoles we used pyrosequencing of the β-tubulin gene (von Samson-Himmelstjerna et al., 2009). In addition, we evaluated changes in the genetics of the nematode population during the course of the replacement period using microsatellite genotyping (Redman et al., 2008, 2015) and examination of β-tubulin haplotypes to measure changes in the population structure. The coupling of these strategies allowed us to not only monitor the changes in drug resistance over time, but also to gain deeper insights into changes occurring at the population genetic level. To the best of our knowledge, the present study is the first to evaluate the resistance status for a longer period of time; we maintained surveillance for 3.5 years post-replacement. Long-term evaluation is necessary to determine if the worm replacement strategy tested was sustainable under the experimental and field conditions used.

## 2 Materials and methods

### 2.1 Experimental design

The timeline of the study is detailed in Table 1. In Spring of 2011, the University of Georgia's sheep flock (Location 1) experienced an outbreak of severe haemonchosis. On the August 3, 2011, lambs (n = 14) and mature sheep (n = 28) were removed from contaminated pastures and housed in pens with concrete floors until September 27, 2011. Sheep were treated orally with a combination of levamisole (Prohibit®, Agri Laboratories, Ltd., St. Joseph, Missouri, USA, 8 mg/kg) and albendazole (Valbazen®, Pfizer Animal Health, Parsippany, New Jersey, USA) on the 3rd, 4th and 5th of August 2011. The initial dose of albendazole was 15 mg/kg, with subsequent doses at 7.5 mg/kg. The mean fecal egg count (FEC) was 1883 eggs per gram (EPG) prior to treatment (August 3, 2011). Fecal egg count reduction (FECR) and associated 95% confidence interval measured on August 15, 2011 was 100.0% (99.1, 100.0). Approximately four weeks after treatment (September 2, 2011), mean fecal egg count (FEC) had increased to 363 eggs per gram (EPG). Sheep were then re-treated using the same 3-day regimen starting on September 12, 2011 which reduced mean FEC by 95% from 363 EPG to 18 EPG (September 21, 2011), representing a 99.1% (97.1, 99.9) FECR from the initial mean FEC of 1883 EPG. Two days later on September 23, 2011, each sheep was infected orally with 5000 third-stage larvae (L3) of UGA-SUSC (George et al., 2018), a laboratory isolate of *H. contortus* that is fully susceptible to avermectin/milbemycins, levamisole, and benzimidazoles. Approximately one week later (September 27, 2011), sheep were moved 17 km to a new property at the UGA Double Bridges Farm (Location 2), that did not previously graze livestock. Prior to the introduction of the sheep, grass washings were conducted according to the methods of Hansen and Perry (1994) to estimate the number of L3 per kg of dry matter present in each of the four paddocks. The number of L3 per kilogram of dry matter recovered from grass washings are presented Table S1 and are likely the result of white-tailed deer that are common in this area. Grazing of paddocks commenced with the least contaminated paddock, paddock 1. At the new property, sheep grazed Max Q fescue grass. Approximately four weeks after being introduced to the new pastures (October 25, 2011), the sheep were evaluated using FAMACHA© (van Wyk and Bath, 2002) and fecal egg counts; none of the adult sheep were treated but all 14 lambs were administered albendazole (7.5 mg/kg). Albendazole was selected as the anthelmintic of choice because the benzimidazole class is the only anthelmintic class with a validated molecular test to detect and measure genotypic changes associated with resistance (von Samson-Himmelstjerna et al., 2009). A primary goal of the present work was to measure changes in resistance to albendazole over time using molecular diagnostic assays including pyrosequence genotyping. Additional treatments with albendazole (7.5 mg/kg) based on FAMACHA© scores of 4 or 5 were administered to 10 sheep on May 3, 2012, and to 27 sheep based on FAMACHA© scores of 3, 4, or 5 on July 7, 2012.

**Table 1:** Timeline of experiment with dates of fecal collections (FECAL), fecal egg count reduction tests (FECRT), larval development assays (LDA), microsatellite genotyping (MSAT), pyrosequence genotyping (PYRO), and sequencing of isotype-1 β-tubulin haplotypes (HAPLO).

Time	Description	FECAL	FECRT	LDA	MSAT	PYRO	HAPLO
Spring 2011	Clinical haemonchosis in flock of sheep						
August 3, 2011	Move flock from contaminated pasture to concrete Drench sheep with levamisole^a^ and albendazole^b^	✓	✓	✓	✓	✓	✓
August 4, 2011	Drench sheep with levamisole^a^ and albendazole^c^						
August 5, 2011	Drench sheep with levamisole^a^ and albendazole^c^						
September 12, 2011	Drench sheep with levamisole^a^ and albendazole^b^						
September 13, 2011	Drench sheep with levamisole^a^ and albendazole^b^						
September 14, 2011	Drench sheep with levamisole^a^ and albendazole^c^						
September 23, 2011	Infect each sheep with 5000 susceptible L3			✓	✓	✓	✓
September 27, 2011	Move sheep to clean pasture						
October 25, 2011	Targeted selective treatment (14 of 42 sheep) with albendazole^c^ using FAMACHA©						
November 10, 2011	1.5 months post-replacement	✓	✓	✓	✓	✓	✓
December 13, 2011	2.5 months post-replacement	✓			✓	✓	
February 1, 2012	4 months post-replacement	✓			✓	✓	✓
May 3, 2012	Targeted selective treatment (10 of 44 sheep) with albendazole^c^ using FAMACHA©	✓					
July 12, 2012	Targeted selective treatment (27 of 46 sheep) with albendazole^c^ using FAMACHA©	✓					
December 4, 2012	14 months post-replacement	✓		✓	✓	✓	✓
April 2, 2013	1.5 years post-replacement	✓	✓	✓	✓	✓	
May 14, 2014	2.5 years post-replacement	✓	✓	✓	✓	✓	✓
May 5, 2015	3.5 years post-replacement	✓	✓	✓	✓	✓	

a 8 mg/kg levamisole.

b 15 mg/kg albendazole.

c 7.5 mg/kg albendazole.

### 2.2 Larval development assay

DrenchRite® Larval Development Assays were performed to evaluate the *in vitro* resistance levels of the *H. contortus* populations throughout the course of the study. The assay was performed in accordance with the manufacturer's guidelines (DrenchRite, Microbial Screening Technologies, Armidale, New South Wales, Australia) with minor modifications as described previously (Kaplan et al., 2007; Howell et al., 2008). This assay evaluates the development of eggs to third-stage larvae in the presence of increasing concentrations of thiabendazole, levamisole, and ivermectin aglycone to provide a measure of susceptibility/resistance to benzimidazoles, imidizothiazoles, and avermectins/milbemycins, respectively.

Drug concentrations were log_10_ transformed prior to analysis. For each well, the percent development to L3 was calculated after correction for development in control wells. Dose response analysis was performed with GraphPad Prism 7.02 (GraphPad Software, La Jolla, California, USA, http://www.graphpad.com) using a variable slope nonlinear regression model. The “log (inhibitor) vs response variable slope (four parameters) logistic equation” was used to generate effective concentration 50 (EC_50_) values with respective 95% confidence intervals. The EC_95_ was also calculated for levamisole and ivermectin aglycone. The x-value for controls were plotted as 0.10 nM for ivermectin, 0.01 μM for levamisole, and 0.003 μM for thiabendazole. The coefficient of determination (R^2^) was reported for each sample. Resistance ratios were calculated as the EC_50_ or EC_95_ of the sample divided by the EC_50_ or EC_95_ of the susceptible isolate (UGA-SUSC). Results for levamisole were also analyzed using a 2-population model in Fit logit (Waller et al., 1985; Dobson et al., 1987) to determine if the population contained a more highly resistant subpopulation and the level of resistance of this subpopulation.

### 2.3 Fecal egg count reduction tests

A fecal egg count reduction test (FECRT) was performed using a label dose of albendazole (7.5 mg/kg) in Fall 2011 (n = 11). In Spring 2013, a FECRT was conducted using label doses of albendazole (7.5 mg/kg, n = 15), ivermectin (Ivermectin Sheep Drench, Durvet, Inc., Blue Springs, Missouri, USA, 200 μg/kg, n = 19), levamisole (8 mg/kg, n = 25), and moxidectin (Cydectin®, Boehringer Ingelheim, St. Joseph, Missouri, USA, 200 μg/kg, n = 31). In Spring 2014, FECRT were conducted with albendazole (n = 12), levamisole (n = 15), moxidectin (n = 16), and a combination of levamisole and moxidectin (n = 9). In Spring 2015, FECRT were conducted with levamisole (n = 17), moxidectin (n = 17), a combination of levamisole and moxidectin (n = 15), and a combination of albendazole, levamisole, and moxidectin (n = 18). All anthelmintics were administered orally at label doses.

FEC were performed using a modified-McMaster technique. The level of detection sensitivity for the modified-McMaster technique was 25 EPG for the majority of tests performed as FECs were very high and sufficient to provide a statistically valid measure of efficacy, however, the 8 EPG method was used to improve the precision of the test as required. The level of detection sensitivity used to complete the pre- and post-treatment counts were consistent within a FECRT. Homogenization of the sample and sodium nitrate solution, slide preparation, and counting were completed as previously described (Noel et al., 2017).

The mean FECR and associated 95% confidence interval were calculated using the web interface of the eggCounts package modified for individual efficacy (http://shiny.math.uzh.ch/user/furrer/shinyas/shiny-eggCounts/) (Wang et al., 2018).

### 2.4 Larval coproculture

Coprocultures were prepared on each sampling date by combining approximately 10.0 g of feces per individual for all individuals in a treatment group that produced sufficient quantity of feces for inclusion. Vermiculite and water were mixed with the pooled feces and incubated at room temperature (approximately 25 °C) for 10–14 days. L3 were recovered with the Baermann technique (Dinaburg, 1942) and phenotypically identified. Greater than 98% of L3 were identified as *H. contortus* at all collections. L3 from 9 collections were stored at −20 °C in water until molecular experiments were completed (Table 1).

### 2.5 Pyrosequence genotyping of single nucleotide polymorphisms associated with benzimidazole resistance in the isotype-1 β-tubulin gene

A 385 bp fragment of the isotype-1 β-tubulin gene containing the position 167 and 200 codons was amplified as previously described (von Samson-Himmelstjerna et al., 2009) in duplicate from two independent lysates of 100 L3 at nine time points throughout the study. Briefly, the forward primer HcPy2PCR For: 5′- GAC GCA TTC ACT TGG AGG AG -3′) and reverse biotinylated primer HcPy2PCR Rev: 5′Biotin- CAT AGG TTG GAT TTG TGA GTT -3′) were used to amplify a 385 bp region of the isotype-1 β-tubulin gene (von Samson-Himmelstjerna et al., 2009). PCR were completed in 50 μL of total volume using the iProof™ High-Fidelity DNA Polymerase Kit (Catalog No. 172-5302, Bio-Rad Laboratories, Inc., Hercules, California, USA) comprised of the following: 10 μL 5x iProof Buffer (1x Final), 1 μL 10 mM dNTP mix (200 μM final concentration), 2.5 μL forward primer (0.5 μM final concentration), 2.5 μL reverse primer (0.5 μM final concentration), 2.0 μL DNA neat lysate, 31.5 μL nuclease free water, and 0.5 μL iProof DNA Polymerase (0.02 U/μL). PCR conditions consisted of an initial denaturation step at 98 °C for 30 s; 35 cycles of the following: denaturation at 98 °C for 10 s, annealing at 43 °C for 30 s, extension at 72 °C 1 min; and a final extension of 72 °C for 7 min. A 2% agarose gel was used to visualize and confirm presence of amplicons of 385 bp in size.

Next, the relative frequency of the F167Y and F200Y SNPs isotype-1 β-tubulin gene were determined using the allele quantification assay available via pyrosequence genotyping (Pyromark Q24, Qiagen, Hilden, Germany). Previously published sequencing primers were used to sequence the F167Y and F200Y mutations: Hc167PySeq1 (5’ –ATA GAA TTA TGG CTT CGT -3′) and Hc200PySeq1 (5’ –TAG AGA ACA CCG ATG AAA CAT-3’) (von Samson-Himmelstjerna et al., 2009). The dispensation order was set at A/TCTCCGTTGTT for the P167 and A/TCTGTATTGAC for the P200. Relative peak heights were used as an approximation of allele frequency for pooled samples as previously validated in pools of adult *H. contortus* (Chaudhry et al., 2015). Plasmids were prepared containing a 385 bp fragment of the isotype-1 β-tubulin gene and with either the wild-type (susceptible) T**T**C sequence or the resistant T**A**C sequence at both the 167 and 200 codon positions. A standard curve was then generated using varying ratios (0:10, 1:9, 3:7, 5:5, 7:3, 9:1, 10:0) of the two plasmids containing the susceptible and resistant genotypes. Measured allele frequencies were corrected based on this standard curve. The standard error of the mean was calculated at each time point.

### 2.6 Microsatellite genotyping and population genetic analysis

A panel of 9 neutral microsatellite loci (Table S2 (Otsen et al., 2000; Redman et al., 2008; Redman et al., 2012; Redman et al., 2015)) were used to genetically differentiate the population at nine time points throughout the study (Table 1). Duplicate lysate pools were used as template for PCR amplification. For each lysate, 100 third-stage larvae were mixed in 100 μL of nuclease free water (Catalog No. W3513, Sigma, Los Angeles, California, USA), 300 μL of DirectPCR (Tail) Lysis Buffer (Catalog No. 101-T, Viagen Biotech, St. Louis, Missouri, USA), and 10 μL Proteinase K (Catalog No. P8107S, New England BioLabs, Inc., Ipswich, Massachusetts, USA) and incubated for 12 h at 55 °C, 1 h at 90 °C, and cooled to 4 °C. PCR amplification was conducted in duplicate to yield a total of four amplicons per time point for each microsatellite. PCR were performed in 10 μL reactions containing 5 μL JumpStart REDTaq ReadyMix Reaction Mix (Catalog No. P0982-800RXN, Sigma, Los Angeles, California, USA), 1 μL of neat lysate, 0.5 μM of each oligonucleotide primer (Integrated DNA Technologies, Skokie, Illinois, USA), and 3 μL of nuclease free water (Catalog No. W3513, Sigma, Los Angeles, California, USA). Thermocycling conditions were 94 °C for 2 min, 40 cycles of 90 °C for 15 s, 54 °C for 30 s, and 72 °C for 1 min, and a final extension step of 72 °C for 7 min. Amplicons were diluted 1:40 in ultrapure water.

Fragment analysis was performed by capillary electrophoresis using an ABI 3730xl sequencer (Applied Biosystems, ThermoFisher Scientific, South San Francisco, California, USA). The internal size standard GeneScan™ 500 ROX™ (Applied Biosystems, ThermoFisher Scientific, South San Francisco, California, USA) was used. Chromatograms were analyzed in Geneious R8 8.0.2 (Biomatters, Ltd., Auckland, New Zealand).

Peak height was used as an estimation of allele frequency (Redman et al., 2008, 2012). The relative percentage of total peak height was calculated for each allele as the height of a single allele divided by the sum of height for all called alleles multiplied by 100. A population of 100 individuals was modeled according to the calculated allele frequency and the principles of Hardy-Weinberg equilibrium. A pairwise population matrix of Nei genetic distance was generated and principal coordinate analysis was performed in GenAlEx version 6.501 (Peakall and Smouse, 2006, 2012).

### *2.7* *Sequencing of isotype-1 β-*tubulin haplotypes

The protocol described above in section 2.6 was used to PCR amplify a 385 bp fragment of the isotype-1 β-tubulin gene encompassing the region containing the three potentially resistance-associated SNPs (F167Y, F200Y, E198A) from lysates of 100 L3 collected at six time points throughout the study (Table 1) with minor modifications described here. The reverse primer Hc BTUB Rev: 5′- CAT AGG TTG GAT TTG TGA GTT -3′ was not biotinylated. PCR conditions consisted of an initial denaturation step at 98 °C for 30 s; 35 cycles of the following: denaturation at 98 °C for 10 s, annealing at 60 °C for 30 s, extension at 72 °C for 30 s; and a final extension of 72 °C for 10 min.

Amplicons were visualized on a 2% agarose gel and cloned directly from purified PCR product (QIAquick® PCR Purification Kit, Catalog No. 28106, Lot No. 145017272, Qiagen, Hilden, Germany). Purified PCR products were cloned using the Zero Blunt® TOPO® PCR Cloning Kit (Catalog No. 450245, Lot No. 1697045, Invitrogen™ by Life Technologies™, Carlsbad, California, USA). Twenty clones per time point (n = 6) were selected and plasmids were purified (GenElute Plasmid Mini Prep Kit, Catalog No. PLN350-1 KT, Lot No. SLBK4190, Sigma, St. Louis, Missouri, USA). Purified plasmid DNA was sequenced using conventional Sanger sequencing in both forward and reverse orientations to create a consensus sequence for each clone.

### 2.8 Bioinformatic and phylogenetic analysis of isotype-1 β-tubulin haplotypes

It is quite likely that single nucleotide polymorphisms that occurred only once in the entire data set of 120 sequences of the isotype 1 *β*-tubulin gene are artifacts due to polymerase induced errors, while single nucleotide polymorphisms appearing more than once in the data set are highly likely to be real polymorphisms (Redman et al., 2015). We therefore removed sequences containing polymerase chain reaction errors from the dataset using bioinformatic filtering of the isotype-1 *β*-tubulin sequences that occurred only once.

A ClustalW alignment of 120 sequences (20 from 6 time points as listed in Table 1) was created in Geneious R8 8.0.2 (Biomatters Ltd., Auckland, New Zealand). A split network (Huson and Bryant, 2006) was generated based on genetic distance using the NeighborNet method (Bryant and Moulton, 2004). Branches with bootstrap support thresholds above 70% were displayed.

## 3 Results

### 3.1 Larval development assay

Prior to replacement, LDA data indicated that the resident population of *H. contortus* at location 1 was highly resistant to both benzimidazole and macrocyclic lactone drugs and susceptible to levamisole; resistance ratios using EC_50_ were 491.00, 0.76, 54.60 for thiabendazole, levamisole, and ivermectin aglycone, respectively (Table 2).

**Table 2:** Larval development assay results.

	Thiabendazole			Levamisole					Ivermectin aglycone				
Time post-replacement	EC^50^ (μM)	Resistance ratio^a^	R^2^	EC^50^ (μM)	Resistance ratio^a^	EC^95^ (μM)	Resistance ratio^b^	R^2^	EC^50^ (nM)	Resistance ratio^a^	EC^95^ (μM)	Resistance ratio^b^	R^2^
UGA-SUSC	0.03	–	0.92	0.93	–	3.87	–	0.96	0.90	–	2.77	–	0.89
Pre-replacement	12.28	491.00	0.86	0.71	0.76	5.40	1.40	0.90	49.10	54.60	645.43	233.01	0.94
1.5 months	0.03	1.18	1.00	0.94	1.01	2.14	0.55	0.97	2.66	2.96	6.09	2.20	0.98
1.5 years	54.91	2195.52	0.78	1.89	2.02	6.57	1.70	0.96	18.44	20.50	88.45	31.93	0.99
2.5 years	28.61	1143.94	0.78	2.73	2.92	38.53	9.96	0.93	26.12	29.04	108.03	39.00	0.97
3.5 years	17.85	713.71	0.85	2.87	3.07	14.69	3.80	0.82	15.99	17.78	488.51	176.36	0.93

a Resistance ratio was calculated as EC50 of tested isolate divided by EC50 of UGA-SUSC.

b Resistance ratio was calculated as EC95 of tested isolate divided by EC95 of UGA-SUSC.

EC_50_ values for thiabendazole indicated that replacement was successful; the post-replacement value was very similar to the susceptible isolate used for replacement (Table 2). However, by 1.5 years post-replacement the population reverted to a very high level of resistance that was maintained throughout the study.

According to the EC_50_, the population was susceptible to levamisole prior to replacement (location1). However, a subpopulation of *H. contortus* that represented 32% of the population prior to replacement was resistant to levamisole. This was reflected by the EC_95_ resistance ratio of 1.40 (Table 2). The EC_95_ was reduced by greater than 50% post-replacement as compared with pre-replacement levels and similar to the susceptible isolate, indicating successful replacement of the majority of the resistant subpopulation. Yet, 1.5 years post-replacement the overall population was resistant to levamisole and there was a highly resistant subpopulation representing 24% of the total population, despite there being no treatments with levamisole administered other than those used in performance of the FECRTs.

For ivermectin aglycone, EC_50_ and EC_95_ values were greatly reduced post-replacement, showing a reduction in resistance ratio of more than 15 and 100 times for the EC_50_ and EC_95_, respectively. However, these values were still two to three times higher than the susceptible isolate used for replacement (Table 2). Similar to other drugs, by 1.5 years post-replacement, LDA indicated a high level of resistance to the macrocyclic lactone class. Additionally, there was a highly resistant subpopulation that continued to increase in level of resistance throughout the 3.5 years post-replacement. These changes occurred despite there being no treatments with avermectin/milbemycin drugs administered other than those used in performance of the FECRTs.

### 3.2 Fecal egg count reduction test

Treatment with albendazole administered seven weeks post-replacement reduced FECs by 98.7% (Table 3), indicating that replacement was successful, which was consistent with results of the LDA. However, by the time efficacy of albendazole was tested again 1.5 years later, the population was highly resistant demonstrating a FECR of only 52.5%. This change in FECR occurred despite the sheep only being treated selectively on three occasions with albendazole (Table 1), with less than 30% of the sheep receiving a treatment. Furthermore, the sheep were not treated with an avermectin/milbemycin during those 1.5 years, however, FECRT indicated substantial resistance to both ivermectin and moxidectin which was again consistent with results of the LDA. In contrast, the population initially remained susceptible to levamisole. Interestingly, one year later (two and a half years post-replacement), results of the FECRT indicated resistance to levamisole, as well as to moxidectin and a combination of both levamisole and moxidectin. Resistance to these same three treatments and a combination of albendazole, moxidectin, and levamisole was present 3.5 years post-replacement (Table 3).

**Table 3:** Mean percent fecal egg count reduction and associated 95% confidence intervals^a^^,^^b^.

Time post-replacement^c^	Albendazole	Ivermectin	Levamisole	Moxidectin	Moxidectin + Levamisole	Albendazole + Moxidectin + Levamisole
1.5 months	98.7 (97.0, 99.4)					
1.5 years	52.5 (33.1, 77.7)	55.1 (36.8, 78.5)	98.8 (96.7, 99.8)	60.7 (48.8, 70.3)		
2.5 years	19.3 (10.0, 39.1)		31.4 (14.8, 52.6)	28.6 (13.3, 50.2)	42.7 (16.4, 67.5)	
3.5 years			23.2 (12.8, 42.9)	22.1 (10.3, 40.6)	62.1 (36.9, 84.8)	67.5 (49.1, 80.4)

a Fecal egg count reduction tests were performed using a modified-McMaster technique using 25 or 8 EPG detection.

b Mean fecal egg count reduction and 95% confidence interval were calculated using the eggCounts package for individual efficacy.

c All drugs and drug combinations were not tested at each time point.

### 3.3 Allele frequency of β-tubulin gene single nucleotide polymorphisms

**Fig. 1:**
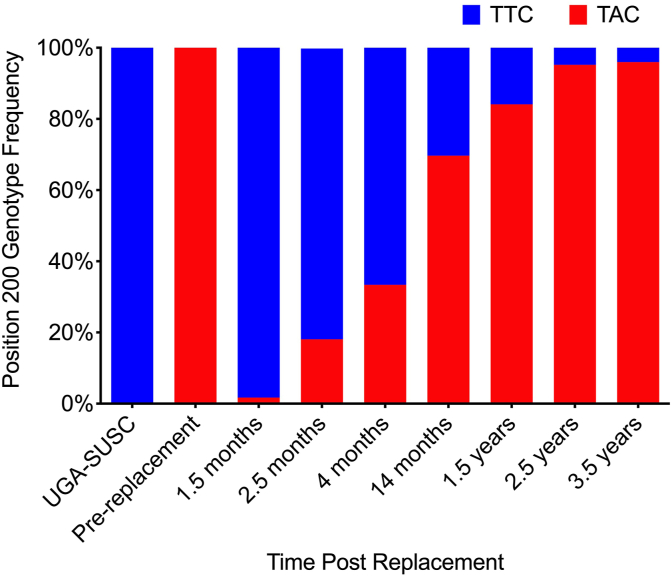
Relative allele frequency of single nucleotide polymorphisms associated with benzimidazole resistance in position 200 of the isotype 1- β-tubulin gene. At 9 time points throughout the study, a region of the isotype-1 β-tubulin gene was PCR amplified from duplicate pools of 100 third-stage larvae. Relative frequency of the F200Y single nucleotide polymorphism was determined using the allele quantification assay available via pyrosequence genotyping. Relative peak heights were used as an approximation of allele frequency for pooled samples. The susceptible genotype (TTC) is shown in blue and resistant genotype (TAC) is shown in red. (For interpretation of the references to colour in this figure legend, the reader is referred to the Web version of this article.)

Prior to replacement, the frequency of the F200Y (T**T**C > T**A**C) and F167Y (T**T**C > T**A**C) resistant polymorphisms in the isotype 1 *β-*tubulin gene were 100.0% and 23.7%, respectively (Fig. 1, Table 4). The susceptible isolate (UGA-SUSC) was comprised almost entirely of the susceptible genotype at both the position 200 and 167 loci (Fig. 1, Table 4). The frequency of resistant genotypes was reduced to 1.7% for the F200Y mutation (Figs. 1) and 0.0% for the F167Y mutation (Table 4) post-replacement. Over the following months, the frequency of the F200Y resistant polymorphism increased steadily to 18.1% and 33.4% at 2.5- and 4-months post-replacement, respectively (Fig. 1). Fourteen months post-replacement the F167Y mutation increased in frequency to 4.1% and then remained at a similar level throughout the duration of the study. However, the F200Y mutation continued to increase in frequency to near fixation by 2.5 years post-replacement (Fig. 1).

**Table 4:** Frequency of resistant isotype-1 β-tubulin genotypes.^a^.

Sample^b^	Position 200 (%TAC)		Position 167 (%TAC)		
	Mean	SE	Mean		SE
UGA-SUSC	0.3	1.2	0.0		0.8
Pre-replacement	100.0	0.3	23.7		1.9
1.5 months	1.7	1.8	0.0		1.4
2.5 months	18.1	2.3	0.0		1.7
4 months	33.4	3.1	0.0		1.7
14 months	69.7	1.3	4.1		2.0
1.5 years	84.1	1.2	5.8		1.4
2.5 years	95.2	0.8	4.5		2.7
3.5 years	96.0	1.5	4.7		0.6

a Relative allele frequency of single nucleotide polymorphisms associated with benzimidazole resistance.

b Time post-replacement.

### 3.4 Microsatellite genotyping

**Fig. 2:**
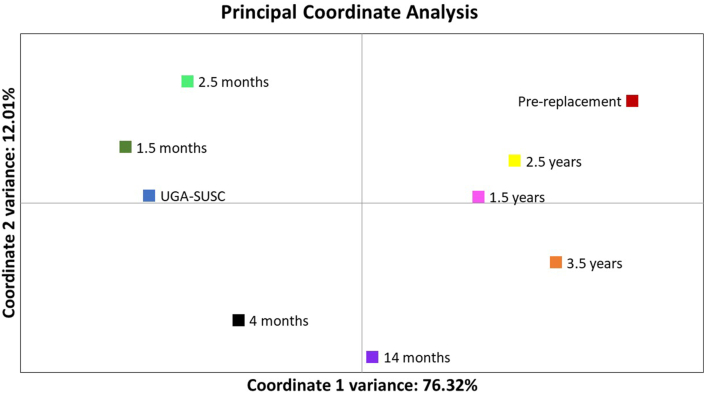
Principal coordinate analysis of microsatellite genotypes. Principal coordinate analysis was performed using GenAlEx 6.501 (Peakall and Smouse, 2006, 2012). A panel of 9 neutral polymorphic microsatellite loci were used to genotype duplicate pools of 100 third-stage larvae at 9 time points throughout the study. Time post-replacement is indicated next to each data point. Peak heights were used as an approximation of allele frequency. The percentage of variation explained by the first two coordinates is shown on the X and Y axes of the graph.

A panel of 9 neutral, polymorphic microsatellite loci were used to describe the genetic relationships between the samples over the course of the study. All of the microsatellites were polymorphic with the total number of alleles per marker varying between two (Hcms40) and fourteen (Hcms25). The principal coordinate analysis was highly informative as it explained 88.33% of variability in the data, 76.32% of variability explained by the x-axis and 12.01% of variability explained by the y-axis (Fig. 2). The resistant population present prior to replacement was the most genetically distant from the susceptible laboratory isolate. For all nine markers, the allele frequencies were similar for the susceptible laboratory isolate and the immediate post-replacement population (1.5 months post-replacement), indicating that these were genetically similar and that replacement was successful. However, over time the population became increasingly similar to the resistant population present prior to replacement. This is evidenced by a shift to the right of the data points in the principal coordinate analysis over time post-replacement (Fig. 2).

### 3.5 Frequency of isotype-1 β-tubulin haplotypes

**Fig. 3:**
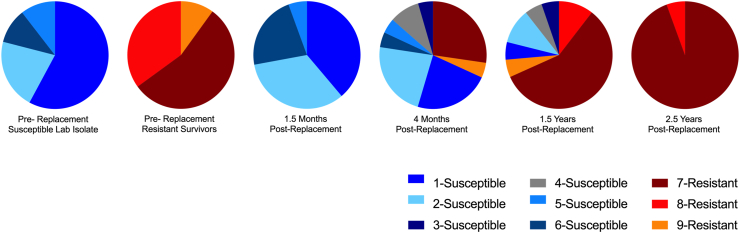
Relative frequency of isotype-1 β-tubulin haplotypes. At 6 time points throughout the study, a region of the isotype-1 β-tubulin gene was PCR amplified from a pool of 100 third-stage larvae, cloned, and 20 clones were sequenced per time point. The relative haplotype frequency at each time point is demonstrated in a pie chart. Blue/gray haplotypes are susceptible at both P167 and P200. Red/orange haplotypes are resistant at P167 and/or P200. (For interpretation of the references to colour in this figure legend, the reader is referred to the Web version of this article.)

Nine haplotypes of the isotype-1 β*-*tubulin gene were identified (Fig. 3 and Supplementary Table 3). Six of the 9 haplotypes contained the susceptible T**T**C genotype at the position 200 and 167 loci and 3 haplotypes contained the resistant T**A**C genotype at the position 200 and/or 167 loci. Overall, the number of haplotypes present initially increased after replacement and then decreased.

The susceptible isolate was comprised of 4 haplotypes, all of which had the T**T**C genotype indicating that all the haplotypes were susceptible to the benzimidazoles. The most common haplotypes in the susceptible isolate were 1 and 2 which were present at 57.9% and 21.1%, respectively. The initial replacement population (1.5 months post-replacement), had a haplotype pattern that was very similar to the susceptible laboratory isolate with haplotypes 1 and 2 again being the most common and present at 38.8% and 33.3%, respectively.

Three haplotypes were present in the initial resistant population (location 1), and all of these carried the resistant T**A**C genotype at the position 200 and/or 167 loci. Two of these haplotypes, 7 and 8 were most common, present at 55.0% and 35.0%, respectively.

In the replaced population, the percentage of resistant haplotypes increased from 0.00% at 1.5 months post-replacement to 31.8% at 4 months and 73.6% at 1.5 years post-replacement, with haplotype 7 being present at 0.0%, 27.3%, and 57.8% at these time points, respectively.

The last haplotype sequencing (2.5 years post-replacement) showed that the population was comprised of 2 resistant haplotypes. Ninety-four point four percent of the sample was comprised of a single resistant haplotype (7), which was also the most common haplotype in the initial resistant population. Haplotypes 1 and 2, the most common susceptible haplotypes in the pre-replacement susceptible laboratory isolate (UGA-SUSC), decreased continuously over the four month and 1.5 year time points, and were completely absent 2.5 years after replacement.

### 3.6 Relatedness of isotype-1 β-tubulin haplotypes

**Fig. 4:**
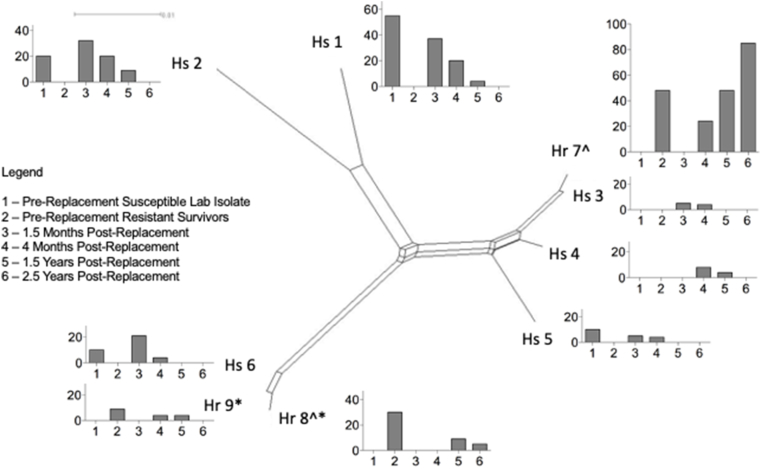
Network analysis of sequences of isotype-1 β-tubulin sequences. A split network was generated with the neighbor-net method of SplitsTree4 to display the relationships between 9 haplotypes that were observed more than once throughout the study over 6 time points. For each haplotype, haplotype frequency (%) per time point is shown on the y axis and time point is shown on the x axis. The six time points can be tracked across the tree as time points are labeled by number. * indicates the F167Y mutation was present. ^ indicates the F200Y mutation was present.

A split network diagram was used to describe the relatedness of the 9 haplotypes that were observed at more than one time point throughout the sampling period (Fig. 4). To provide a visual display of changes in the frequency of each haplotype over time, the percentage of the population comprised of each haplotype at the 6 time points tested are displayed in bar graphs (Fig. 4).

The nine haplotypes segregated into three clusters (Fig. 4). Haplotypes 1 and 2, which both contain the susceptible T**T**C genotype at the position 200 and 167 loci, were phylogenetically very similar and were present in highest frequencies in the susceptible isolate and immediately post-replacement. However, the other two clusters in Fig. 4 contain both susceptible and resistant genotypes.

The two resistant haplotypes which occurred at multiple time points in the study and contained the F167Y mutation, haplotypes 8 and 9, were present prior to replacement but decreased initially post-replacement. Although resistant haplotype 8 which contains both the F167Y and F200Y resistance associate polymorphisms was the second most common haplotype prior to replacement (location 1), haplotype 8 was not detected in the replaced population until 1.5 years post-replacement, and then remained low through 2.5 years post-replacement. In contrast, the only F200Y resistant haplotype that occurred at multiple time points in the study, and did not contain the F167Y mutation, haplotype 7, increased in frequency towards fixation following replacement.

## 4 Discussion and conclusions

The present study evaluated changes in drug susceptibility and population genetic structure following replacement of a multiple-anthelmintic resistant population of *H. contortus* with a susceptible laboratory isolate on a sheep farm. While the strategy of replacement has previously been tested as a methodology to replace resistant alleles with susceptible alleles in gastrointestinal nematodes of sheep (Van Wyk and Van Schalkwyk, 1990; Bird et al., 2001; Aumont, 2002; Sissay et al., 2006; Moussavou-Boussougou et al., 2007; Muchiut et al., 2019), this is the first time this strategy has been evaluated for a period greater than 18 months post-replacement. Additionally, this study is the first to use both population genetics analyses and measurement of molecular markers for resistance following replacement. Combining these data with *in vivo* and *in vitro* phenotypic data enabled us to not only precisely measure the changes in drug susceptibility over time, but also explain these changes at the population genetic level.

We show using multiple phenotypic and genetic measures that the initial multiple-anthelmintic resistant population was successfully replaced with a susceptible laboratory isolate. Resistance ratios based on LDA EC_50_ were reduced from 491.00 to 1.18 for thiabendazole and from 54.60 to 2.96 for ivermectin aglycone (Table 2). Treatment with albendazole reduced FEC by 98.7% (Table 3) and the frequency of the F200Y SNP associated with benzimidazole resistance was reduced from 100.0% to 1.7% (Fig. 1, Table 4). Aumont et al. (2002), Bird et al. (2001), and Sissay et al. (2006) also reported reversion to susceptibility based on FECRT results, however egg reduction was the only measurement used, which is rather insensitive (Martin et al., 1989), and follow-up analysis was conducted for only a short period of time post-replacement. Using controlled efficacy tests and egg hatch assays, Van Wyk and Van Schalkwyk (1990) reported reversion to susceptibility in three out of five test paddocks following introduction of a susceptible isolate. Using strategic and highly effective anthelmintic treatments combined with a large number of seeder lambs infected with a susceptible strain of *H. contortus,* over two years Michuit et al. (2019) successfully replaced a highly benzimidazole-resistant population of *H. contortus* on a paddock with a susceptible strain of *H. contortus* as evidenced by controlled efficacy tests performed 16 months post-replacement. Using controlled efficacy tests, fecal egg count reduction tests, egg hatch assays, and genetic markers of benzimidazole resistance, Moussavou-Boussougou et al. (2007) also reported reversion to susceptibility, however follow-up analysis was only conducted 4 months post-replacement. Additionally, Moussavou-Boussougou et al. (2007) reported approximately 20% of benzimidazole-resistance alleles in the post-replacement population which is more than 10 times the level detected in the present study.

It is notable that replacement is relative, not absolute, and is based on the principle of dilution. We were unable to completely remove the initial resistant population with the treatment regimen used; this result should be expected most often given that replacing a highly multiple-anthelmintic resistant population is a challenging endeavor. Despite reducing the mean initial FEC by greater than 99%, sheep still had a mean FEC of 18 eggs per gram following two rounds of treatment. The first round of treatment included three consecutive days of a combination treatment including levamisole and albendazole which reduced the mean FEC from 1883 EPG to 0 EPG. Levamisole was selected as the primary drug for removal of the initial population as the larval development assay results indicated the population was susceptible to levamisole. Albendazole was included to further improve the efficacy of the combination treatment as it is the most potent of the benzimidazole anthelmintics. However, the mean FEC increased to 363 EPG four weeks post-treatment, despite the sheep being housed on concrete floors to prevent further ingestion of infective larvae. We believe this rise in FECs was due to the development of immature larvae that survived the initial levamisole and albendazole treatment regimen. It is well established that levamisole has reduced efficacy against larval stages of *Haemonchus contortus* and thus very possible that these larval stages survived the treatment, developed to adults, mated, and produced eggs within four weeks post-treatment. Alternative drugs such as monepantel and derquantel that may have been more effective are not approved in the USA, and thus were not an option for us to use.

These results and the lack of highly effective anthelmintics demonstrate the difficulty in removing multiple-drug resistant populations of nematodes which is particularly important in an experiment to replace a population. Hence, a second round of treatment including three consecutive days of a combination treatment of levamisole and albendazole was required to eliminate the adults that developed in the 40 days following the initial round of treatment and were producing these eggs. The second round of treatment reduced mean FEC from 1883 EPG to 18 EPG, representing 99.1% FECR overall. Overall, these data indicate that our protocol was quite successful in reducing and diluting the benzimidazole-resistant alleles in the population. Given the subsequent results, it seems likely that most of the few remaining nematodes shedding these eggs were highly resistant to benzimidazoles, and possibly also to levamisole, the two drugs used for treatment. Consequently, despite replacing the majority of the resistant *H. contortus* population, the sheep remained infected with a small number of highly-resistant worms.

After confirming that we had successfully replaced the resistant population, we treated only with albendazole, and treatments were applied infrequently, and selectively using FAMACHA (van Wyk and Bath, 2002). Albendazole was selected as the treatment of choice because there is a well-validated molecular test to measure genotypic changes associated with resistance to benzimidazoles and a primary goal of the present work was to measure these changes. Thus, we expected resistance to benzimidazoles to develop slowly over time, and we aimed to monitor the dynamics of changes in susceptibility by making both phenotypic and genotypic measurements. However, surprisingly, when we retested the population using FECRT 1.5 years post-replacement, we found that there was reversion to resistance not only to albendazole, but also to ivermectin, and moxidectin (Table 2, Table 3, Table 4). We knew from previous experiments that the initial multi-drug resistant population on the original sheep farm (location 1) was highly resistant to both ivermectin and moxidectin. Given this prior knowledge, and the new data, it is highly likely that the ‘resistant survivors’ carried from location 1 were highly resistant to both ivermectin and moxidectin, since reversion to resistance occurred despite no treatments being administered with these drugs. We hypothesized that the small population of ‘resistant survivors’ rapidly increased in relative frequency compared with the susceptible replaced population following the move to location 2 for reasons unrelated to treatment, leading to the rapid reversion to resistance of the overall population. This led us to examine the population genetics in greater detail in order to gain deeper insights into why this rapid reversion occurred.

The primary resistant isotype-1 β-tubulin haplotype prior to replacement (haplotype 7) was also the most common resistant haplotype following development of resistance at time points of 4 months, 1.5 years, and 2.5 years (Fig. 4, Table S3). These results provide strong genetic evidence the initial ‘resistant survivors’ increased in frequency over time post-replacement. The population genetics work also clearly shows that the resistant population that emerged after replacement is comprised of the three haplotypes present in the resistant population prior to replacement. These results provide evidence that the larvae that were identified on the pasture at location 2 (Table S1) and any potential contamination of *H. contortus* by wild deer did not influence the overall population dynamics nor contribute to the final resistant population that emerged. It is possible that haplotypes 3 and 4 that were first identified 4 months post-replacement and not identified in the susceptible isolate or resistant population prior to replacement represent the L3 on pasture at location 2 at the time of the grass washings, however, these two haplotypes reduced in frequency over time and did not contribute to the final resistant population. Thus, the level of pasture contamination present at location 2 prior to replacement had no impact on the population dynamics over time.

The low number of anthelmintic treatments that were applied using targeted-selective treatment and the high level of refugia maintained on pasture and in untreated animals would not be expected to place high drug-selection pressure on the population. Specifically, a total of three selective treatments were applied over 10 months where 33% of sheep were treated in October 2011, 23% of sheep were treated in May 2012, and 59% of sheep were treated in July 2012. These were the only treatments administered over 18 months. The weather conditions during the Spring and Summer treatments were high rainfall, high humidity, and warm temperatures which promote development of *H, contortus* larvae and hence a high level of refugia on pasture. Barnes et al. (1995) used computer modelling to determine that treating 80% of animals while leaving 20% untreated delayed the development of resistance. Dobson et al. (2011) showed that leaving 10% of animals untreated delayed the development of resistance by 35% of the time which suggests the percentage of animals left untreated in the present study delayed the development of resistance. Hence, there is sufficient evidence that the level of drug selection pressure in the present study was low and was not suspected to influence the population dynamics and changes in resistance seen in the present study. Therefore, we believe resistance developed due to reasons unrelated to anthelmintic treatment.

Though it is not possible to prove without performing a new large and detailed study, it seems very likely that field-fitness of UGA-SUSC, which was a laboratory isolate, was impaired, and did not compete well with the resistant sub-population. This reduction in the level of fitness in UGA-SUSC may be associated with the methodology used to cycle laboratory isolates. UGA-SUSC was cryopreserved for many years prior to being passaged through lambs or kids. We received the isolate after passage had been performed for several generations, and we do not have information on the number of L3 that were used to establish the infection in the initial passage. Subsequent passages were made with only a few thousand L3 that developed in coproculture under highly controlled laboratory conditions. Under these conditions there is no selection for environmental fitness in the next generation, and each passage creates a genetic bottleneck and can further reduce the diversity of the isolate. This contrasts greatly to a field situation where L3 must develop in a sometimes hostile and changing environment, thousands of worms are infecting numerous animals in a pasture environment, tens to hundreds of millions of eggs are shed onto the pasture each day, and transmission with new parasites is constantly occurring in a dynamic fashion. Thus, one would expect field isolates to be superior in fitness and diversity under natural environmental conditions as compared with laboratory isolates. Specifically, the resistant population remaining prior to replacement evolved under natural environmental conditions in Athens, Georgia, and thus would be expected to have much greater environmental fitness than a lab population that was maintained under highly controlled and optimal conditions.

Four haplotypes of the isotype 1 *β-tubulin* gene were identified in the susceptible laboratory isolate (UGA-SUSC) and three haplotypes were identified in the resistant population prior to replacement. Redman et al. (2015) surveyed 7 seven sheep farms in the UK with *H. contortus* and reported between one to eight haplotypes in this gene per farm. Thus, the number of haplotypes identified in UGA-SUSC and the resistant survivors prior to replacement are consistent with those previously reported in other *H. contortus* field populations. As would be expected, the number of haplotypes identified in the isotype 1 *β-tubulin* gene was highest at 4 months and 1.5 years post-replacement, as ‘resistant survivors’ and UGA-SUSC worms mated and reproduced under minimal drug selection pressure. However, the relatedness of these haplotypes is also important to describe the diversity in the isotype 1 *β-tubulin* gene throughout the course of replacement. Haplotypes 1 and 2 were present at 57.9% and 21.1%, respectively, in UGA-SUSC and clustered together in the network analysis (Fig. 4), suggesting these haplotypes were closely related and the laboratory isolate lacked diversity. However, the ‘resistant survivors’ contained haplotypes 7 and 8 at 55.0% and 35.0%, respectively. Haplotypes 7 and 8 were more distantly related on the network analysis (Fig. 4) suggesting that this field population was more diverse.

Interestingly, resistant haplotypes 8 and 9 that both contain the F167Y mutation decreased over time following replacement; whereas resistant haplotype 7 that did not contain the F167Y mutation increased in frequency towards fixation following replacement. These results suggest that these haplotypes varied in levels of fitness and may suggest that haplotypes that contain the F167Y mutation are less fit than those that contain the F200Y mutation. This observation is interesting given that the F200Y resistance polymorphism is much more common than the F167Y mutation in multiple species, populations and countries (Chaudhry et al., 2014, 2015).

In the present study, successful replacement of a resistant population of *H. contortus* with a susceptible population did not result in long term maintenance of drug efficacy. However, we feel that a flaw in our methodology, rather than a flawed strategy likely was responsible. In the course of our genetic analyses performed following completion of the field phase of the study, we demonstrated that UGA-SUSC lacked genetic diversity. Additionally, we did not ‘field-adapt’ the isolate prior to using it to infect the sheep. Given our results, it seems quite plausible that UGA-SUSC was “unfit” for survival in the natural world, and was not able to adequately survive and reproduce in the environmental conditions in Athens, Georgia. We believe that parasite replacement likely remains a viable strategy for enhanced parasite control on farms, but that great care is needed when selecting the methodology to use. Parasite populations must have sufficient fitness to survive under local conditions to be effectively used in replacement strategies. If we were to attempt parasite replacement again, we would first field-adapt the susceptible parasite population over a lengthy period so the parasites would undergo natural environmental selection over several life-cycles of infection, and be required to survive and reproduce under natural field conditions of the local climate. In conclusion, the present study demonstrates the experimental power of combining molecular, *in* vitro, and *in vivo* assays to describe phenotypic and genotypic changes in field populations of nematodes. Combining measurement of changes occurring on the population genetic level with phenotypic data can provide a valuable tool for understanding the impact of control strategies on parasite populations.

## Declaration of competing interest

The authors declare that they have no known competing financial interests or personal relationships that could have appeared to influence the work reported in this paper.

## References

[bib1] Aumont G., Chevalier M., Hostache G., Mandonnet N. (2002). Substitution du peuplement helminthique en élevage caprin viande en milieu tropical humide: Une technique pour maintenir la diversité des populations parasitaires et contrôler les résistances aux anti-parasitaires.

[bib2] Barnes E.H., Dobson R.J., Barger I.A. (1995). Worm control and anthelmintic resistance: adventures with a model. Parasitol. Today.

[bib3] Bird J., Shulaw W.P., Pope W.F., Bremer C.A. (2001). Control of anthelmintic resistant endoparasites in a commercial sheep flock through parasite community replacement. Vet. Parasitol..

[bib4] Bryant D., Moulton V. (2004). Neighbor-Net: an agglomerative method for the construction of phylogenetic networks. Mol. Biol. Evol..

[bib5] Chaudhry U., Miller M., Yazwinski T., Kaplan R., Gilleard J. (2014). The presence of benzimidazole resistance mutations in Haemonchus placei from US cattle. Vet. Parasitol..

[bib6] Chaudhry U., Redman E.M., Raman M., Gilleard J.S. (2015). Genetic evidence for the spread of a benzimidazole resistance mutation across southern India from a single origin in the parasitic nematode Haemonchus contortus. Int. J. Parasitol..

[bib7] Dinaburg A.G. (1942). The efficiency of the Baermann apparatus in the recovery of larvae of Haemonchus contortus. J. Parasitol..

[bib8] Dobson R.J., Griffiths D.A., Donald A.D., Waller P.J. (1987). A genetic model describing the evolution of levamisole resistance in trichostrongylus colubriformis , a nematode parasite of sheep. Math. Med. Biol..

[bib9] Dobson R.J., Barnes E.H., Tyrrell K.L., Hosking B.C., Larsen J.W.A., Besier R.B., Love S., Rolfe P.F., Bailey J.N. (2011). A multi‐species model to assess the effect of refugia on worm control and anthelmintic resistance in sheep grazing systems. Aust. Vet. J..

[bib10] George M.M., Lopez-Soberal L., Storey B.E., Howell S.B., Kaplan R.M. (2018). Motility in the L3 stage is a poor phenotype for detecting and measuring resistance to avermectin/milbemycin drugs in gastrointestinal nematodes of livestock. Int. J. Parasitol. Drugs Drug Resist..

[bib11] Gilleard J., Devaney E. (2013). Haemonchus contortus as a paradigm and model to study anthelmintic drug resistance. Parasitology.

[bib12] Hansen J., Perry B. (1994). The Epidemiology, Diagnosis and Control of Helminth Parasites of Ruminants.

[bib13] Herrera-Manzanilla F.A., Ojeda-Robertos N.F., González-Garduño R., Cámara-Sarmiento R., Torres-Acosta J.F.J. (2017). Gastrointestinal nematode populations with multiple anthelmintic resistance in sheep farms from the hot humid tropics of Mexico. Vet. Parasitol. Reg. Stud. Rep..

[bib14] Howell S.B., Kaplan R.M., Williamson L.H., Burke J.M., Miller J.E., Terrill T.H., Valencia E., Williams M.J., Zajac A.M. (2008). Prevalence of anthelmintic resistance on sheep and goat farms in the southeastern United States. J. Am. Vet. Med. Assoc..

[bib15] Howell S.B., Storey B.E., Collins J.B., Kaplan R.M. (2017). A 16-year Retrospective Analysis of Anthelmintic Resistance on Small Ruminant Farms in the United States.

[bib16] Huson D., Bryant D. (2006). Application of phylogenetic networks in evolutionary studies. Mol. Biol. Evol..

[bib17] Kaplan R.M., Vidyashankar A.N. (2012). An inconvenient truth: global worming and anthelmintic resistance. Vet. Parasitol..

[bib18] Kaplan R.M., Vidyashankar A.N., Howell S.B., Neiss J.M., Williamson L.H., Terrill T.H. (2007). A novel approach for combining the use of in vitro and in vivo data to measure and detect emerging moxidectin resistance in gastrointestinal nematodes of goats. Int. J. Parasitol..

[bib19] Leathwick D.M., Besier R.B. (2014). The management of anthelmintic resistance in grazing ruminants in Australasia--strategies and experiences. Vet. Parasitol..

[bib20] Leathwick D.M., Ganesh S., Waghorn T.S. (2015). Evidence for reversion towards anthelmintic susceptibility in Teladorsagia circumcincta in response to resistance management programmes. Int. J. Parasitol. Drugs Drug Resist..

[bib21] Leathwick D.M., Waghorn T.S., Miller C.M., Candy P.M., Oliver A.M.B. (2012). Managing anthelmintic resistance – use of a combination anthelmintic and leaving some lambs untreated to slow the development of resistance to ivermectin. Vet. Parasitol..

[bib22] Martin P.J., Anderson N., Jarrett R.G. (1989). Detecting benzimidazole resistance with faecal egg count reduction tests and in vitro assays. Aust. Vet. J.; ISSN.

[bib23] Martínez-Valladares M., Martínez-Pérez J.M., Robles-Pérez D., Cordero-Pérez C., Famularo M.R., Fernández-Pato N., Castañón-Ordóñez L., Rojo-Vázquez F.A. (2013). The present status of anthelmintic resistance in gastrointestinal nematode infections of sheep in the northwest of Spain by in vivo and in vitro techniques. Vet. Parasitol..

[bib24] Moussavou-Boussougou M.N., Silvestre A., Cortet J., Sauve C., Cabaret J. (2007). Substitution of benzimidazole-resistant nematodes for susceptible nematodes in grazing lambs. Parasitology.

[bib25] Muchiut S.M., Fernández A.S., Steffan P.E., Riva E., Fiel C.A. (2018). Anthelmintic resistance: management of parasite refugia for Haemonchus contortus through the replacement of resistant with susceptible populations. Vet. Parasitol..

[bib26] Muchiut S.M., Fernández A.S., Lloberas M., Steffan P.E., Luque S.E., Cardozo P.A., Bernat G.A., Riva E., Fiel C.A. (2019). Recovery of fenbendazole efficacy on resistant Haemonchus contortus by management of parasite refugia and population replacement. Vet. Parasitol..

[bib27] Noel M.L., Scare J.A., Bellaw J.L., Nielsen M.K. (2017). Accuracy and precision of mini-FLOTAC and McMaster techniques for determining equine strongyle egg counts. J. Equine Vet. Sci..

[bib28] Otsen M., Plas M.E., Lenstra J.A., Roos M.H., Hoekstra R. (2000). Microsatellite diversity of isolates of the parasitic nematode Haemonchus contortus. Mol. Biochem. Parasitol..

[bib29] Peakall R., Smouse P.E. (2006). Genalex 6: genetic analysis in Excel. Population genetic software for teaching and research. Mol. Ecol. Notes.

[bib30] Peakall R., Smouse P.E. (2012). GenAlEx 6.5: genetic analysis in Excel. Population genetic software for teaching and research—an update. Bioinformatics.

[bib31] Playford M., Smith A., Love S., Besier R., Kluver P., Bailey J. (2014). Prevalence and severity of anthelmintic resistance in ovine gastrointestinal nematodes in Australia (2009–2012. Aust. Vet. J..

[bib32] Redman E., Packard E., Grillo V., Smith J., Jackson F., Gilleard J.S. (2008). Microsatellite analysis reveals marked genetic differentiation between Haemonchus contortus laboratory isolates and provides a rapid system of genetic fingerprinting. Int. J. Parasitol..

[bib33] Redman E., Sargison N., Whitelaw F., Jackson F., Morrison A., Bartley D.J., Gilleard J.S. (2012). Introgression of ivermectin resistance genes into a susceptible Haemonchus contortus strain by multiple backcrossing (backcrossing Haemonchus contortus). PLoS Pathog..

[bib34] Redman E., Whitelaw F., Tait A., Burgess C., Bartley Y., Skuce P.J., Jackson F., Gilleard J.S. (2015). The emergence of resistance to the benzimidazole anthlemintics in parasitic nematodes of livestock is characterised by multiple independent hard and soft selective sweeps. PLoS Neglected Trop. Dis..

[bib35] Sissay M.M., Asefa A., Uggla A., Waller P.J. (2006). Anthelmintic resistance of nematode parasites of small ruminants in eastern Ethiopia: exploitation of refugia to restore anthelmintic efficacy. Vet. Parasitol..

[bib36] Torres-Acosta J.F.J., Mendoza-de-Gives P., Aguilar-Caballero A.J., Cuéllar-Ordaz J.A. (2012). Anthelmintic resistance in sheep farms: update of the situation in the American continent. Vet. Parasitol..

[bib37] van Wyk J.A., Bath G.F. (2002). The FAMACHA system for managing haemonchosis in sheep and goats by clinically identifying individual animals for treatment. Vet. Res..

[bib38] van Wyk J.A., Hoste H., Kaplan R.M., Besier R.B. (2006). Targeted selective treatment for worm management—how do we sell rational programs to farmers?. Vet. Parasitol..

[bib39] Van Wyk J.A., Van Schalkwyk P.C. (1990). A novel approach to the control of anthelmintic-resistant Haemonchus contortus in sheep. Vet. Parasitol..

[bib41] von Samson-Himmelstjerna G., Walsh T.K., Donnan A.A., CarriÈRe S., Jackson F., Skuce P.J., Rohn K., Wolstenholme A.J. (2009). Molecular detection of benzimidazole resistance in Haemonchus contortus using real-time PCR and pyrosequencing. Parasitology.

[bib42] Waller P.J., Dobson R.J., Donald A.D., Griffiths D.A., Smith E.F. (1985). Selection studies on anthelmintic resistant and susceptible populations of Trichostrongylus colubriformis of sheep. Int. J. Parasitol..

[bib43] Wang C., Torgerson P.R., Kaplan R.M., George M.M., Furrer R. (2018). Modelling anthelmintic resistance by extending eggCounts package to allow individual efficacy. Int. J. Parasitol. Drugs Drug Resist..

